# Dissociating compatibility effects and distractor costs in the additional singleton paradigm

**DOI:** 10.3389/fpsyg.2013.00434

**Published:** 2013-07-17

**Authors:** Charles L. Folk

**Affiliations:** Department of Psychology, Villanova UniversityVillanova, PA, USA

**Keywords:** attentional capture, compatibility effects, top-down control, additional singleton paradigm, load theory

## Abstract

The interpretation of identity compatibility effects associated with irrelevant items outside the nominal focus of attention has fueled much of the debate over early versus late selection and perceptual load theory. However, compatibility effects have also played a role in the debate over the extent to which the involuntary allocation of spatial attention (i.e., attentional capture) is completely stimulus-driven or whether it is contingent on top-down control settings. For example, in the context of the *additional singleton paradigm*, irrelevant color singletons have been found to produce not only an overall cost in search performance but also significant compatibility effects. This combination of search costs and compatibility effects has been taken as evidence that spatial attention is indeed allocated in a bottom-up fashion to the salient but irrelevant singletons. However, it is possible that compatibility effects in the additional singleton paradigm reflect parallel processing of identity associated with low perceptual load rather than an involuntary shift of spatial attention. In the present experiments, manipulations of load were incorporated into the traditional additional singleton paradigm. Under low-load conditions, both search costs and compatibility effects were obtained, replicating previous studies. Under high-load conditions, search costs were still present, but compatibility effects were eliminated. This dissociation suggests that the costs associated with irrelevant singletons may reflect filtering processes rather than the allocation of spatial attention.

## INTRODUCTION

Selective visual attention is a construct invoked to account for the fact that only a fraction of the information contained in the retinal image is processed to the point of influencing goal-oriented behavior. A controversial issue in research on visual attention concerns the point in the stream of visual information processing at which the system selects from the available information the subset that is passed on for further processing. Historically, this issue has been framed in terms of whether selection occurs before or after the processing of stimulus identity, with the former referred to as *early selection* and the latter as *late selection* (Broadbent, 1958; Deutsch and Deutsch, 1963; Treisman, 1969; Allport, 1977; Treisman and Gelade, 1980).

One approach to determining the locus of selection has been to evaluate the influence of the identity of stimuli that appear outside the nominal focus of attention. For example, in the classic *flankers task* pioneered by Eriksen and Eriksen (1974), observers respond to the identity of an attended letter at fixation, and the response compatibility of letters appearing outside the focus of attention is manipulated. The presence of *compatibility effects* in this paradigm has been taken as evidence that the processing of letter identity is not dependent on the prior allocation of attention (i.e., a late locus of selection). Miller (1987), for example, found that even when flanking letters never appeared as targets (and were therefore completely task-irrelevant), they still produced response compatibility effects when their appearance was correlated with particular targets. Others, however, have argued that flanker compatibility effects do, in fact, depend on the prior allocation of attentional resources (i.e., an early locus of selection). For example, Eriksen and Eriksen (1974) interpreted their original results as evidence for limitations in the ability to restrict the allocation of attention to the central letter. Yantis and Johnston (1990) found that although relatively simple, two-letter, arrays produced standard flanker effects, these effects were eliminated when displays increased to eight items. The authors suggested that flanker effects associated with simple display are the result of unfocused spatial attention that “spills over” onto irrelevant stimuli. In contrast, “cluttered” displays encourage more tightly focused attention, eliminating flanker effects. This proposal is consistent with a more recent theoretical treatment known as *load theory*, proposes that the apparent locus of selection depends on the interaction between the resources required to efficiently perform the task (i.e., load) and the resources available to do so (Yantis and Johnston, 1990; Lavie, 1995). Specifically, when the resource load of the central, focused-attention task is low, the available resources are not fully consumed, and the remaining resources are passively and automatically allocated to other items in the display, resulting in the processing of the identity of those items. When the resource load of the focused-attention task is high, there are no remaining resources available for the processing of irrelevant display information. In support of this notion, Lavie and her colleagues have shown that compatibility effects associated with irrelevant peripheral stimuli are indeed eliminated if the perceptual difficulty of the central task is increased (Lavie and Tsal, 1994; Lavie, 1995, 2000, 2005; Lavie and Cox, 1997; Lavie and Fox, 2000; but see Tsal and Benoni, 2010).

In addition to their role in the debate over the locus of attentional selection, compatibility effects have also played a role in a long standing debate over the degree to which irrelevant but salient stimuli involuntarily elicit shifts of spatial attention, a phenomenon referred to as *attentional capture*. On one side of the debate is the “pure-capture” perspective, according to which preattentive processing results in the purely bottom-up or stimulus-driven allocation of attention that is completely impervious to top-down attention set or behavioral goals (Theeuwes, 1992, 1994, 1995, 2010). On the other side of the debate is the “contingent capture” perspective, according to which attentional capture is dependent on whether the capturing stimulus carries properties that match the task-related top-down “set” of the observer (Folk et al., 1992, 1994, 2002; Folk and Remington, 1998; Wyble et al., 2013).

One of the strongest pieces of evidence in support of the pure-capture perspective comes from the “additional singleton” paradigm. In the typical task, participants search for a singleton target defined on one feature dimension and response time to discriminate the orientation of a line segment inside the target is measured as a function of whether an “additional singleton,” defined on a different feature dimension, is also present in the display or not. For example, Theeuwes (1992) had subjects search for a singleton target defined as a green diamond among a variable number of green circles. The presence of an additional, irrelevant, color singleton distractor produced a significant cost in response time, and the magnitude of this effect was dependent on the salience of the distractor relative to the target. Given that participants knew the defining feature of the target (shape) with complete certainty, they should have been able to instantiate a selective top-down set for that feature. Thus, the fact that a salient distractor defined in an orthogonal feature dimension (color) produced a cost in performance is consistent with a model in which the allocation of spatial attention is driven entirely by bottom-up salience, independent of top-down set.

More importantly for the present purposes, Theeuwes (1995) used a compatibility manipulation to provide converging evidence for the capture of spatial attention by irrelevant, additional singletons. The same method as Theeuwes (1992) was employed, but the identity of the element inside the distractor singleton was systematically varied, such that half the time it was the same as the element in the target singleton, and half the time it was the identity of the other possible target element. The presence of the additional singleton produced both search costs as well as significant compatibility effects associated with the identity of the element inside that irrelevant singleton. The combination of these two effects was interpreted as strong evidence for shifts of spatial attention to the location of the distractor singleton that occur independent of top-down set.

However, several alternative accounts of the additional singleton effect have been proposed. For example, Bacon and Egeth (1994) have argued that although the costs associated with the presence of irrelevant singletons reflect shifts of spatial attention, those shifts reflect the adoption of a top-down “singleton search mode” in which the system is set for singletons in general. This is in contrast to “feature search mode” in which the system is set for particular feature values. In support of this claim, Bacon and Egeth (1994) showed that when participants are forced to look for a target defined by a specific feature (e.g., when the target shape appears among heterogeneous non-target shapes), the effect of irrelevant singletons is eliminated (see also Leber and Egeth, 2006; but see Theeuwes, 2004).

Another interpretation of the irrelevant singleton effect attributes distractor costs not to the capture of spatial attention, but to a form of non-spatial competition known as “filtering costs” (Kahneman et al., 1983; Folk and Remington, 1998). According to this account, when a distractor is present, preattentive segmentation of the display results in two objects that “pop-out” from the background elements (i.e., the target and the distractor, by virtue of their singleton status). In contrast, when no distractor is present, only one object (the target) pops out from the background. Thus, according to the filtering cost explanation, the increase in response time associated with the presence of an irrelevant distractor reflects a delay in the allocation of spatial attention as the system resolves the competition between the two objects with respect to which should be the recipient of an attentional shift.

However, if, as proposed by the filtering costs account, focal attention is not shifted to the location of the irrelevant singleton, then why does the identity of the distractor produce compatibility effects as in Theeuwes (1995)? One possibility is that the preattentive segmentation of the search displays into two objects (i.e., the target and distractor), results in a stimulus that not only requires a time-consuming filtering operation, but can be characterized as a “low-load” display. Consistent with this possibility, Lavie and Cox (1997) found that load is associated not with the total number of stimuli on the display but with the number of *salient* stimuli in the display. This finding suggests that the effective set-size (and therefore load) in the irrelevant singleton paradigm is determined by the number of singletons. Thus, according to load theory, even if focal attention is shifted directly to the target singleton after the filtering operation is complete, given that the effective set-size is two, there may be enough attentional capacity left over to allow the automatic, parallel processing of the target and distractor identities, resulting in both filtering costs and distractor compatibility effects on response time.

There is already evidence that manipulations of processing load can influence the degree to which irrelevant singletons produce compatibility effects, at least in serial visual search (as opposed to parallel feature search in the typical additional singleton paradigm). Theeuwes and Burger (1998) found that when serial search of a display is required to detect a target letter, the presence of a non-target color singleton distractor produced compatibility effects if there was uncertainty about the particular color assignment on a given trial. The authors concluded that the presence of compatibility effects shows that in the absence of top-down expectations regarding target and distractor colors, singleton distractors can capture attention even during serial visual search. However, when Gibson and Bryant (2008) added a load (set-size) manipulation to the same task, compatibility effects associated with the color singleton distractor were eliminated with increases in perceptual load. The authors concluded that rather than reflecting attentional capture, the compatibility effects reported by Theeuwes and Burger (1998) were due to the passive, parallel allocation of unconsumed resources to the processing of the singleton letter.

There is also evidence from other paradigms that when only two display elements are presented (i.e., under low-load conditions), items that are known to appear outside the focus of spatial attention can nonetheless produce significant compatibility effects. For example, Folk et al. (2009) used a spatial cuing task in which letters appeared inside boxes to the left and right of fixation. One of the letters (the target) was red and the other was white. In addition, on half the trials the identity of the non-target letter was compatible with the target, and on the other trials it was incompatible. The search display was preceded by a cue display in which sets of four, abruptly onset dots appeared around each of the boxes, with one set of dots (the cue) appearing in red and the other in white. The location of the cue was non-predictive of the subsequent target location. Significant cuing effects were obtained, confirming that attention had been captured to the location of the cue. A significant effect of compatibility was also obtained. Most importantly, compatibility effects were obtained even on valid cue trials. Thus, even when the attention was allocated to the location of the target (as independently confirmed by the cuing effect), the identity of the unattended non-target letter on the other side of fixation produced compatibility effects. The authors concluded that even when spatial attention is focused at one location, parallel processing of the identity of irrelevant information can occur under low-load conditions.

In summary, there is evidence from serial search tasks and spatial cuing experiments that compatibility effects associated with unattended stimuli can emerge under low-load conditions and that these effects reflect parallel processing associated with excess processing capacity. These studies call into question whether the compatibility effects associated with distractors in the additional singleton paradigm necessarily reflect the allocation of spatial attention, or whether they are the result of parallel processing under low processing load.

The present experiments were designed to determine the nature of the distractor compatibility effects found in the additional singleton paradigm by introducing a processing load manipulation. If, as argued by Theeuwes and colleagues, compatibility effects in this paradigm provide converging evidence for the capture of spatial attention by the singleton distractor, then they should always co-vary with the costs associated with the presence of the distractor. That is, if a distractor produces a cost in search time, it should also produce a compatibility effect because distractor costs are assumed to reflect the allocation of spatial attention to the distractor location. If, however, compatibility effects reflect parallel processing of target and distractor identity associated with “low-load” displays, and these effects are functionally distinct from the costs produced by the presence of a distractor (e.g., filtering costs), then it should be possible to dissociate distractor costs from compatibility effects. Specifically, if the perceptual load is increased, then compatibility effects should be selectively reduced or eliminated, leaving the costs associated with the presence of the distractor intact.

Perceptual load is typically manipulated by varying display size. However, in the additional singleton paradigm, the target and distractor are both singletons, and therefore “pop-out” independent of display size. Indeed, as discussed above, this feature of the additional singleton paradigm is what renders the displays “low load,” in that regardless of display size, the displays are perceptually segmented into one or two objects (depending on whether a distractor is present or not). Therefore, in the present experiments perceptual load was instead manipulated by varying whether responses were contingent on the presence of a particular conjunction of features across feature dimensions (i.e., color and shape; see Lavie, 1995). In order to accomplish this, we used a version of the additional singleton paradigm in which the target and distractor singletons were both defined within the same feature dimension (color), but different with respect to the particular feature value (target singletons were green and distractor singletons were red). In Experiment 1, we first show that the typical additional-singleton effects can be obtained using such within-dimension singletons. Experiment 2 added the critical perceptual load manipulation. Specifically, the shapes of the display elements were varied between circles and squares and in the critical “high-load” condition, responses to the green target were contingent on the green singleton also being a circle; responses were to be withheld from a green square. Lavie (1995) has previously shown that this type of perceptual load manipulation can eliminate compatibility effects associated with non-target distractors in a flankers task. It is also important to note that this means of manipulating perceptual load involves no changes to the physical properties of the display between low- and high-load conditions. Therefore, according to the pure-capture perspective, it should have no effect on the bottom-up salience of the distractor, and consequently no influence on whether the singleton distractor captures attention. To anticipate the results, although the presence of a distractor produced a search cost regardless of load condition, significant compatibility effects were only obtained in the low-load condition. Experiment 3 shows that the elimination of compatibility effects under high load is not simply the result of an overall increase in response times associated with high-load conditions.

## EXPERIMENT 1

**FIGURE 1:**
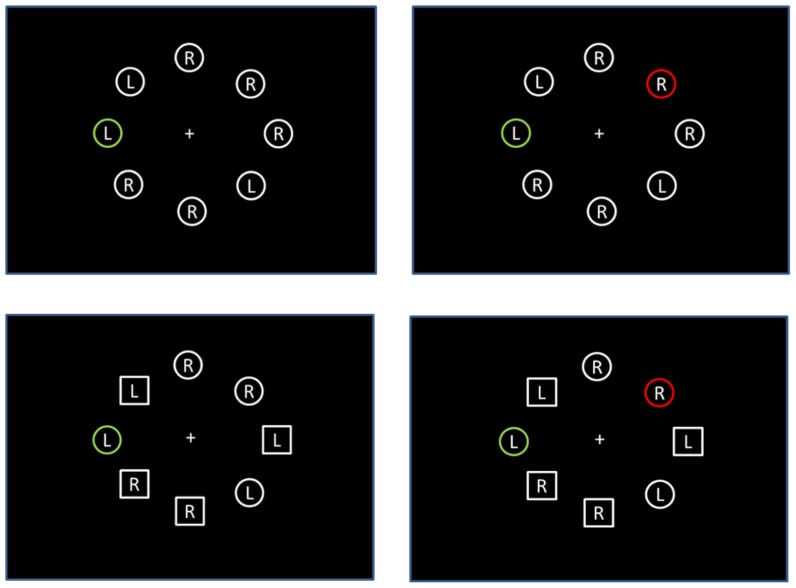
** Examples of displays from Experiments 1 (top row) and 2 (bottom row)**.

Given that the additional singleton paradigm has traditionally involved target and distractor singletons defined across different feature dimensions (e.g., shape target paired with a color distractor), the first experiment was conducted to be sure that the basic additional-singleton and compatibility effects can be found using within-dimension color singletons defined by different colors. Specifically, participants searched for a green circle among white circles and responded to the identity of the letter (R or L) inside the green circle. On half the trials an additional red circle distractor appeared and the compatibility of the letter inside distractor, relative to the letter inside the target, was varied across trials (see **Figure 1**,top row).

### MATERIALS AND METHODS

#### Participants

Twenty undergraduates from Villanova University participated in partial fulfillment of a course requirement. Participants ranged in age from 18 to 20 years, and all were tested for normal or corrected-to-normal binocular near visual acuity (20/30 or better) and normal color vision using a Titmus II vision tester.

#### Apparatus

Stimuli were generated and responses collected by a Zenith 386 microcomputer equipped with a Sigma Design, Color 400 (680 × 400) graphics board. Stimuli were displayed on a Princeton Graphics Systems Ultrasync monitor. The monitor was placed in an enclosed viewing box at a distance of 50 cm.

#### Stimuli

Search displays consisted of either six or eight white (RGB: 255, 255, 255; CIE: *x* = 0.35, *y* = 0.36) circles (1.15° in diameter) equally spaced on the circumference of an imaginary circle (8.2° in diameter) centered on a white fixation cross (0.34° × 0.34°). Each circle contained either the letter “R” or the letter “L” (0.75° × 0.9°) displayed in white. On no-distractor trials one of the circles (i.e., the target) was green (RGB: 85, 255, 85; CIE: *x* = 0.33, *y* = 0.55). On distractor trials, in addition to the green target, one other circle (the distractor) was red (RGB: 255, 85, 85; CIE: *x* = 0.56, *y* = 0.34).

#### Design

An experimental session consisted of 5 blocks of 48 trials. Half the trials in each block contained six circles and half contained eight. In addition, for half the trials the green target circle contained the letter “R” and for half the letter “L.” On distractor trials, the red circle contained the same letter as the target (compatible trials) on half the trials and the other letter on the other half of trials (incompatible trials). The identity of the letters in the other circles was determined randomly on each trial. The positions of the target and distractor were also determined randomly on each trial.

#### Procedure

The experimental session lasted approximately 1 h. Subjects were instructed to respond as quickly and accurately as possible, and to maintain fixation on the central fixation cross throughout each trial. Subjects were also fully informed with respect to the irrelevance of the distractors, and were encouraged to “ignore the distractor if possible.”

The trial sequence began with the presentation of the fixation cross for 1 s. The cross then blinked off for 250 ms as a warning signal that the trial was beginning. The search display appeared 500 ms later and remained on the screen until the participant responded, at which point all stimuli were removed from the screen.

Subjects responded to target trials by pressing the “0” key on the numeric keypad of the computer keyboard with the forefinger of the their right hand if the letter inside the green target was an “R” and the left forefinger of the left hand if the letter inside the target was an “L.” Response time was measured from the onset of the target display. Incorrect responses elicited a 500 ms, 1000-Hz computer tone, and were followed by a “buffer” trial with parameters drawn randomly from the set for that block. Response times for error and buffer trials were not included in the data analysis.

### RESULTS

**FIGURE 2:**
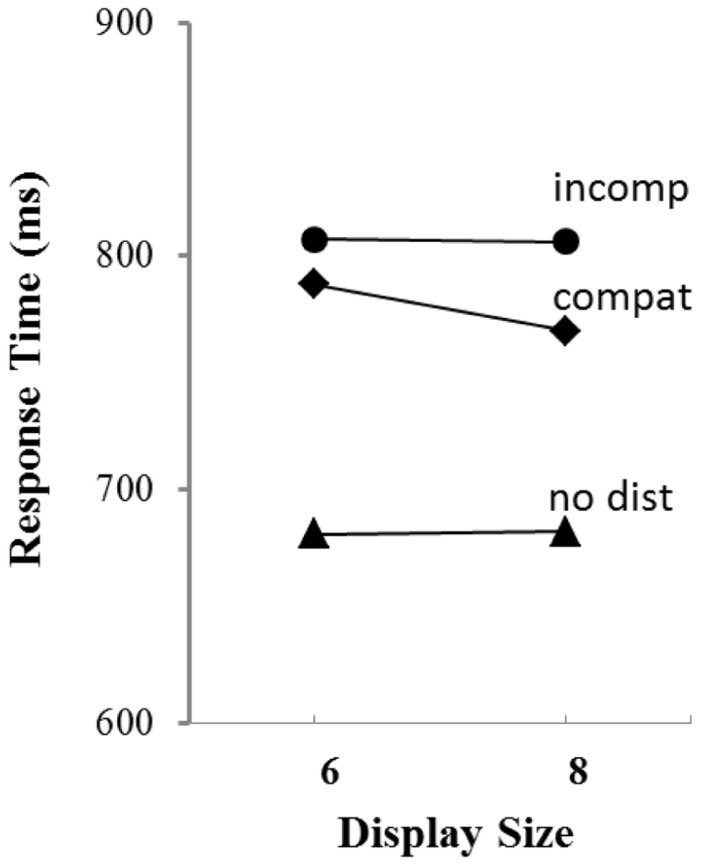
** Mean response time as a function of display size and distractor type in Experiment 1**.

Response times as a function of display size and distractor condition are shown in **Figure 2** and error rates are reported in **Table 1**. The data were subjected to a 2 × 3 repeated measures ANOVA with display size (6, 8) and distractor condition (no distractor, compatible distractor, incompatible distractor) as factors. As is evident in the figure, the presence of a distractor produced a cost in response time that was confirmed by a significant main effect of distractor condition, *F*(2,38) = 32.35, MSE = 5296, *p* < 0.0001. Neither the main effect of display size nor the interaction were significant, *F *< 1 for both. To determine if the compatibility of the distractor influenced response time, a separate repeated measures ANOVA was conducted on just those trials containing a distractor, with compatibility and display size as factors. Only the main effect of compatibility was significant, *F*(1,19) = 4.89, MSE = 3315, *p* < 0.05.

**Table 1:** Mean error rate as a function of distractor condition and display size for Experiments 1, 2a, 2b, and 3.

	Display size							
	Experiment 1		Experiment 2a		Experiment 2b		Experiment 3	
	6	8	6	8	6	8	6	8
**Distractor condition**
No distractor	0.011	0.014	0.016	0.024	0.008	0.008	0.019	0.022
Compatible distractor	0.003	0.007	0.008	0.014	0.006	0.005	0.018	0.020
Incompatible distractor	0.011	0.012	0.018	0.019	0.004	0.003	0.025	0.021

Overall mean error rate was less than 1%. An ANOVA of the error data with display size and distractor as factors yielded only a main effect of distractor condition, *F*(1,19) = 3.37, MSE = 1.94, *p* < 0.05. As is evident in **Table 1**, this effect is associated with lower error rates in the compatible distractor condition.

### DISCUSSION

The results of the first experiment show that when searching for a singleton of a specific color, the presence of additional singleton of a different color produces both a search cost as well as a compatibility effect. Having replicated the basic additional-singleton effects in the context of color singleton displays, we are now ready to institute variations in perceptual load by incorporating variations in the shape of the display elements and manipulating whether responses are contingent on a particular combination of color and shape (high load) or not (low load).

## EXPERIMENT 2a AND 2b

Experiment 2 was similar to Experiment 1, with the exception that within each search display, half the elements were circles and half were squares. In Experiment 2a, the task was exactly the same as Experiment 1; participants responded to the identity of the letter inside the green item (regardless of whether it was a circle or square). In Experiment 2b, the displays were exactly the same, but participants were instructed to only respond if the green element was a circle; they were to withhold a response if the green element was a square (see **Figure 1**, bottom row).

### MATERIALS AND METHODS

#### Participants

Twenty-four Villanova University undergraduates participated, 12 in Experiment 2a and 12 in Experiment 2b. All participated in partial fulfillment of a course requirement. Participants ranged in age from 18 to 20 years, and all were tested for normal or corrected-to-normal binocular near visual acuity (20/30 or better) and normal color vision using a Titmus II vision tester.

#### Apparatus

The apparatus was the same as Experiment 1.

#### Stimuli

Stimuli were the same as in Experiment 1, with the exception that half the elements in each display were circles and half were squares (1.5° × 1.5°). Green targets and red distractors could be either a circle or square.

#### Design

The design was similar to Experiment 1, except the number of blocks was increased to 8. In addition, within each block, the green target was a circle on two-thirds (32) of the trials and a square on one-third (16) of the trials. The red distractor was equally likely to be circle or a square.

#### Procedure

The procedure was identical to Experiment 1, with the exception that in Experiment 2b, subjects were instructed to only respond to the identity of the green target if it was a square. Otherwise, they were to withhold a response.

### RESULTS

#### Experiment 2a

Response times as a function of display size and distractor condition are shown in left panel of **Figure 2** and error rates are reported in **Table 1**. The data were subjected to a 2 × 3 repeated measures ANOVA with display size (6, 8) and distractor condition (no distractor, compatible distractor, incompatible distractor) as factors. As in Experiment 1, the presence of a distractor produced a cost in response time that was confirmed by a significant main effect of distractor condition, *F*(2,22) = 25.85, MSE = 2580, *p* < 0.0001. The main effect of display size was not significant, *F*(1,11) = 2.26, MSE = 1008, *p* > 0.10, but the interaction was significant, *F*(2,22) = 4.48, MSE = 381, *p* < 0.05. To determine if the compatibility of the distractor influenced response time, a separate repeated measures ANOVA was conducted on just those trials containing a distractor, with compatibility and display size as factors. Only the main effect of compatibility was significant, *F*(1,11) = 22.48, MSE = 17072, *p* < 0.0001.

Overall mean error rate was less than 2%. An ANOVA of the error data with display size and distractor as factors yielded no significant effects.

#### Experiment 2b

Response times as a function of display size and distractor condition are shown in the middle panel of **Figure 2** and error rates are reported in **Table 1**. The data were subjected to a 2 × 3 repeated measures ANOVA with display size (6, 8) and distractor condition (no distractor, compatible distractor, incompatible distractor) as factors. Once again, the presence of a distractor produced a cost in response time that was confirmed by a significant main effect of distractor condition, *F*(2,22) = 219.77, MSE = 1977, *p* < 0.0001. The main effect of display size was not significant, nor was the interaction, *F* < 1 for both. To determine if the compatibility of the distractor influenced response time, a separate repeated measures ANOVA was conducted on just those trials containing a distractor, with compatibility and display size as factors. Unlike Experiment 2a, the effect of compatibility was not significant, *F*(1,11) = 0.08, MSE = 1676, *p* > 0.05. The effect of display size and the interaction were also non-significant, *F* < 1 for both.

Overall mean error rate was less than 1%. An ANOVA of the error data with display size and distractor as factors yielded no significant effects.

#### Comparison of compatibility effects for Experiments 2a and 2b

To directly compare the impact of distractor compatibility under the different load conditions of Experiments 2a and 2b, the data from the conditions in which a distractor appeared were entered into a mixed factor ANOVA with Experiment (2a vs. 2b) as the between-subjects variable, and display size and compatibility as the within-subjects variables. The analysis yielded a significant main effect of compatibility, *F*(1,22) = 12.22, MSE = 1614, *p* < 0.01. Crucially, the interaction was also significant, *F*(2,22) = 9.48, MSE = 1614, *p* < 0.01, confirming that the manipulation of load across Experiments 2a and 2b significantly modulated the influence of distractor compatibility.

### DISCUSSION

The results of Experiment 2 provide strong evidence that the compatibility effects associated with distractors in the additional singleton paradigm can be dissociated from the search costs produced by those same distractors. Specifically, although the displays were exactly the same in Experiments 2a and 2b, increasing the perceptual load by conditionalizing responses on a conjunction of color and shape in 2b completely eliminated the compatibility effect while leaving search costs intact. This pattern is inconsistent with the claim that the combination of search costs and compatibility effects constitute converging evidence for the capture of spatial attention by singleton distractors. If search costs reflect the allocation of spatial attention to the distractor, then the presence of such costs should always be associated with the presence of compatibility effects because attention has been allocated to the distractor letter. The fact that high perceptual load eliminated compatibility effects while leaving search costs intact is, however, consistent with the hypothesis that compatibility effects are produced by parallel processing of target and distractor in low-load displays, whereas search costs reflect delays associated with filtering processes (which should be unaffected by perceptual load).

It is important to point out, however, that the elimination of compatibility effects in the high-load condition was accompanied by an overall increase in response times, and are therefore confounded with overall task difficulty. It is possible that compatibility effects were generated even in the high-load conditions, but the overall increase in processing time allowed the effects to dissipate by the time response selection occurred. One way to rule out this possibility is to show that in low-load conditions, which we *know* produce compatibility effects (such as those in Experiment 1), the effects are still present when overall response times are increased. However, one must be careful that the manipulation used to increase overall response times does not itself affect perceptual load nor interfere with response selection. Therefore, Experiment 3 replicated the low perceptual load conditions of Experiment 1, but replaced the “R’s” and “L’s” with rotated “T’s” and “L’s.” It was assumed that using rotated T’s and L’s would increase task difficulty by requiring the insertion of an additional mental process (mental rotation) that is associated with central processing resources and therefore does not increase perceptual load or interfere with response selection. Support for this assumption comes from Ruthruff et al. (1995) who using a psychological refractory period paradigm, showed that mental rotation requires central, rather than perceptual, processing resources. The general logic for addressing the task difficulty confound is similar to Lavie and DeFokert (2003) who showed that compatibility effects associated with low perceptual load conditions were still evident when general task difficulty was increased through sensory degradation. If compatibility effects associated with singleton distractors are simply be “hidden” by long overall response times, then assuming the rotation manipulation is successful, the compatibility effects found in Experiment 2 should not be evident in Experiment 3. If, however, compatibility effects do not dissipate with increases in overall response time, then the results should be similar to those found in Experiment 1.

## EXPERIMENT 3

### MATERIALS AND METHODS

#### Participants

Twenty Villanova University undergraduates participated in partial fulfillment of a course requirement. Participants ranged in age from 18 to 20 years, and all were tested for normal or corrected-to-normal binocular near visual acuity (20/30 or better) and normal color vision using a Titmus II vision tester.

#### Apparatus

The apparatus was the same as Experiment 1.

#### Stimuli

Stimuli were the same as in Experiment 1, with the exception that the “R’s” and “L’s” were replaced with “T’s” and “L’s” whose rotation with respect to vertical was chosen randomly from among 0°, 90°, 180°, and 270°.

#### Design and procedure

The design and procedure were identical to Experiment 1, with the exception that participants were instructed to press the “0” key on the numeric keypad of the computer keyboard with the forefinger of the their right hand if the letter inside the green target was an “T” and the left forefinger of the left hand if the letter inside the target was an “L.”

### RESULTS

**FIGURE 3:**
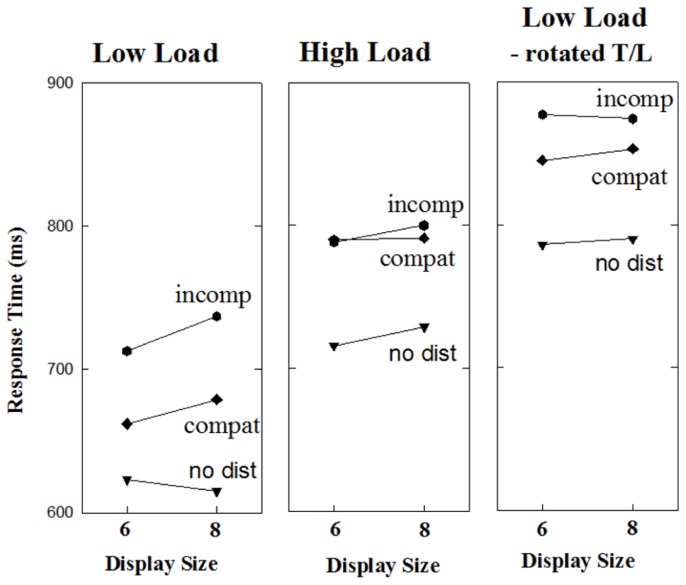
** Mean response time as a function of display size and distractor type in Experiments 2a (right panel), 2b (middle panel), and 3 (right panel)**.

Response times as a function of display size and distractor condition are shown in the right panel of **Figure 3** and error rates are reported in **Table 1**. The data were subjected to a 2 × 3 repeated measures ANOVA with display size (6, 8) and distractor condition (no distractor, compatible distractor, incompatible distractor) as factors. The only significant effect was a main effect of distractor condition, *F*(2,38) = 48.68, MSE = 1637, *p* < 0.0001. To determine if the compatibility of the distractor influenced response time, a separate repeated measures ANOVA was conducted on just those trials containing a distractor, with compatibility and display size as factors. Only the main effect of compatibility was significant, *F*(1,19) = 20.21, MSE = 716, *p* < 0.0001.

Overall mean error rate was less than 3%. An ANOVA of the error data with display size and distractor as factors yielded no significant effects.

### DISCUSSION

As is evident in **Figure 3**, the present study was successful with respect to increasing overall response times. Most importantly, the compatibility effect remained intact even with overall response times similar to those found in Experiment 2b. Thus, it is reasonable to conclude that the lack of compatibility effects in Experiment 2b is not due to the dissipation of such effects with increased response times, but rather reflects the elimination of compatibility effects under high perceptual load. This strengthens the conclusion that compatibility effects can be dissociated from search costs in the additional singleton paradigm, and calls into question the claim that compatibility effects and search costs provide converging evidence for the capture of spatial attention by singleton distractors.

## GENERAL DISCUSSION

Manipulations of the response compatibility of task-irrelevant stimuli have played an important role in the development of theories of selective attention. With respect to the present studies, such compatibility effects have been used to infer whether salient stimuli elicit involuntary shifts of spatial attention that are independent of top-down set. Specifically, the presence of both compatibility effects and search costs in the additional singleton paradigm have been interpreted as converging evidence for the purely bottom-up capture of attention by salient singletons (Theeuwes, 1996). However, evidence from other paradigms suggests that under low perceptual load conditions, the presence of compatibility effects can be dissociated from shifts of spatial attention, reflecting instead the parallel processing of the identity of task-irrelevant distractors due to availability of residual attentional capacity (Gibson and Bryant, 2008; Folk et al., 2009).

The present experiments were conducted to determine whether the compatibility effects found in the additional singleton paradigm reflect shifts of spatial attention, or parallel processing under low-load conditions. Experiment 1 replicated the basic additional singleton effect, showing that even when target and distractor singletons are defined by specific color values with the color dimension, the presence of a distractor singleton produces both search costs and compatibility effects. Experiments 2a and 2b introduced a load manipulation in which responses were (2b) or were not (2a) conditionalized on a particular combination of color and shape. Previous research using a flankers task has shown that this form of perceptual load manipulation modulates compatibility effects from irrelevant flankers. It was hypothesized that if search costs and compatibility effects provide convergent evidence for shifts of spatial attention, then they should both obtain regardless of perceptual load, because the bottom-up salience of the distractor does not change across load conditions, and any stimulus that captures attention should produce a compatibility effect. If, however, compatibility effects reflect parallel processing under low-load conditions rather than a shift of spatial attention, then they should decrease with increasing perceptual load while leaving search costs intact.

The results of Experiment 2 show that under low-load conditions, both search costs and compatibility effects were present, but under high-load conditions, only search costs were present. Experiment 3 confirmed that this effect is not simply due to the increase in overall response times, as search costs and compatibility effects were obtained for low-load displays in which overall response times were increased by increasing the time required to identify the target. Thus, the elimination of compatibility effects under high-load conditions in Experiment 2 suggests that rather than reflecting shifts of spatial attention, compatibility effects in this paradigm are due to parallel processing of the target and distractor when there is excess attentional capacity (i.e., under low-load conditions). Moreover, the clear dissociation between search costs and compatibility effects also calls into question whether search costs reflect shifts of spatial attention, since any stimulus to which spatial attention is directed should produce compatibility effects.

### ALTERNATIVE INTERPRETATIONS

It is important, however, to consider other possible interpretations of the influence of load in the present experiments. For example, Belopolsky et al. (2007) have shown that whether a singleton distractor captures attention is dependent on the size of the “attentional window,” which can be influenced by the difficulty of search. Specifically, increases in search difficulty require a smaller attentional window, resulting in a serial search strategy that eliminates capture. Thus, perhaps the high-load condition of Experiment 2 results in a smaller attentional window which prevents capture and therefore eliminates the compatibility effect. This possibility can be ruled out, however, because the presence of the distractor continued to produce search costs even in the high-load condition.

Another possibility is that our assumption about the asymmetric relationship between attention shifts and compatibility effects is wrong. We have assumed that if spatial attention is shifted to a stimulus it will always produce compatibility effects, whereas compatibility effects can obtain even in the absence of a shift of spatial attention (due, for example, to parallel processing under low-load conditions). However, logically, the absence of compatibility effects does not necessarily imply the absence of an attentional shift. For example, perhaps the singleton distractor does capture attention, but under high-load conditions attentional disengagement is so fast that the identity of the distractor is not processed (Theeuwes, 2010). Although logically possible, it is difficult to imagine how changing the response requirements for identical displays would result in changes in the speed of disengagement. In both conditions, the participants know that “red” is not the target color, and it is not clear why attention should be disengaged more rapidly when responses to the green target are contingent on its shape. Indeed, one might expect that when the task requires the consideration of shape as well as color, attention might tend to linger even longer on any given singleton.

Finally, one might also question whether our assumptions underlying the logic of Experiment 3 are valid. We assumed that the insertion of a mental rotation operation would increase task difficulty, and thereby lengthen overall response time, without affecting perceptual load or response selection. The fact that compatibility effects were still obtained under such an increase in difficulty was taken as evidence that the lack of compatibility effects in the critical high-load condition of Experiment 2b was not simply the result of the dissipation of the effect with longer response times. However, one might question whether the mental rotation required in Experiment 3 was as independent of response and perceptual processes as we assumed (Band and Miller, 1997; Heil et al., 1999; Pannebakker et al., 2011). For example, Pannebakker et al. (2011) found evidence that mental rotation can interfere with shifts of spatial attention. More importantly, Band and Miller (1997) found that mental rotation produced interference in response preparation. Thus, if the elimination of compatibility effects in Experiment 2 reflects response selection processes that, when given enough time, can counteract the activation of incompatible responses, then the mental rotation required in Experiment 3 might have interfered with those processes such that incompatible response activation could not be counteracted, even with longer overall response times. The present data cannot definitively rule out this alternative interpretation.

### SPATIAL SHIFTS OR FILTERING COSTS?

The present results show that compatibility effects in the additional singleton paradigm can be influenced by perceptual load and can therefore be dissociated from search costs. As argued above, this dissociation implies that search costs produced by singleton distractors do not reflect the capture of spatial attention because if attention is allocated to the distractor, then the identity of the character at the distractor location should be processed, resulting in compatibility effects. We argue that the pattern of results in the present experiments is uniquely consistent with a filtering cost interpretation. According to this account, preattentive segregation of the typical additional singleton display results in the pop-out of the distractor and target. This has two dissociable consequences. First, when a singleton is present, it produces a competition with the target for the allocation of attention, which is ultimately resolved in the target’s favor by virtue of a bias associated with the top-down set for the target color. We assume this competitive filtering process does not require resources, but does take time to complete. Thus, the presence of a distractor singleton produces a search cost that is not influenced by perceptual load. The second consequence is that the pop-out of the target and distractor singletons reduces the effective set-size of the search display to two, which can be characterized as a low-load display. Thus, even though focal attention is shifted only to the target, there are residual perceptual resources that they are allocated in parallel to the singleton distractor, resulting in the processing of its identity and the production of compatibility effects. When the perceptual resource requirements are increased in the high-load conditions, there are no longer residual resources available for distractor processing.

The conclusion that search costs in the additional singleton paradigm do not reflect shifts of spatial attention is consistent with several recent studies using event-related potential (ERP) measures (McDonald et al., 2013). For example, Jannati et al. (2013) measured ERP components associated with attention allocation (N2pc) and attentional suppression (P_D_) while participants completed an additional singleton task in which the target singleton was defined by a fixed shape and the distractor singleton a fixed color. The presence of a distractor singleton produced a search cost relative to no distractor trials, replicating the standard additional-singleton effect. However, the ERP analysis showed that the salient distractor did not elicit an N2pc, but did elicit a P_D_ on fast-response trials. In addition, the target singleton did elicit an N2pc whose timing was unaffected by the presence of the salient distractor. The authors concluded that salient singletons in the additional singleton paradigm do not elicit shifts of attention, but do produce a time-consuming competition for attention that is resolved by suppressing the distractor location.

### PERCEPTUAL LOAD OR DILUTION?

We have argued that the compatibility effects associated with distractor singletons in the additional singleton paradigm can be accounted for in terms of load theory (Lavie, 1995). However, Tsal and Benoni (2010) have recently argued that what appear to be perceptual load effects may actually reflect the “dilution” of perceptual encoding. According to this view, when “perceptual load” in a flankers task is manipulated by increasing display size (i.e., adding “neutral” letters), the elimination of flanker compatibility effects may result from the dilution of the flanker representations by the neutral letters rather than the unavailability of residual perceptual processing resources. This is because the processing of neutral letters activates feature detectors that would otherwise be devoted to the encoding of the irrelevant flanker, thereby diluting its effect. Consistent with this account, several studies have shown that flanker compatibility effects are eliminated under low-load but high-dilution conditions (Benoni and Tsal, 2010; Tsal and Benoni, 2010; Wilson et al., 2011). In the present experiments, however, the manipulation of processing load involved a change in the complexity of the perceptual operations required rather than any change in the properties of the displays. Thus, the degree of potential dilution (i.e., the degree to which feature detectors associated with letter identification are activated) was held constant across the low- and high-load conditions of Experiment 2. This suggests that the modulation of compatibility effects in the present experiments most likely reflects true load effects rather than dilution. It is important to point out, however, that regardless of the specific mechanism, the critical finding in the present studies is the dissociation between search costs and compatibility effects, which, as argued above, suggests that search costs do not reflect the capture of spatial attention.

## CONCLUSION

The present experiments are the first to document a dissociation between search costs and compatibility effects in the additional singleton paradigm. Specifically, increasing perceptual load by conditionalizing responses on a conjunction of color and shape eliminated distractor compatibility effects while leaving distractor search costs intact. This pattern suggests that distractor compatibility effects in the additional singleton paradigm are the result of automatic, parallel identity processing in low-load displays. The results also highlight the fact that caution must be exercised in the interpretation of distractor compatibility effects in attentional capture paradigms, in that distractor compatibility effects can reflect processes other than shifts of spatial attention. This is not to say that compatibility effects can never be diagnostic of attention shifts, but that in order to conclusively tie compatibility effects to attentional capture, one must show that they covary with other, independent, measures of capture. For example, using a spatial cuing paradigm, Folk and Remington (2006) found compatibility effects associated with the presentation of spatial cues, but only when those cues also produced cuing effects indicative of an attentional shift to the cue.

Finally, the dissociation between search costs and compatibility effects in the present experiments suggests that the search costs in the additional singleton paradigm also do not reflect the capture of spatial attention. Specifically, if the costs were due to a shift of attention to the cue, then compatibility effects should have been present regardless of perceptual load. Therefore, the results undermine the claim that the additional singleton effect is strong evidence for the notion that attention allocation is driven solely by the bottom-salience of display elements. There is one final caveat, however. The present experiments explored the effects of perceptual load for singletons defined within the color dimension. Thus, additional research is needed to determine if the load effects found in the current experiments will generalize to additional singleton paradigms in which the target and distractor singletons are defined across dimensions (e.g., shape and color), or when the singleton distractor is defined by other stimulus properties such as abrupt onset (e.g., Schreij et al., 2008).

## Conflict of Interest Statement

The author declares that the research was conducted in the absence of any commercial or financial relationships that could be construed as a potential conflict of interest.
